# Author Correction: Atomically dispersed cobalt catalyst anchored on nitrogen-doped carbon nanosheets for lithium-oxygen batteries

**DOI:** 10.1038/s41467-022-33297-7

**Published:** 2022-09-29

**Authors:** Peng Wang, Yingying Ren, Rutao Wang, Peng Zhang, Mingjie Ding, Caixia Li, Danyang Zhao, Zhao Qian, Zhiwei Zhang, Luyuan Zhang, Longwei Yin

**Affiliations:** Key Laboratory for Liquid-Solid Structural Evolution and Processing of Materials, Ministry of Education, School of Materials Science and Engineering, Shandong University, Jinan, 250061 PR China

**Keywords:** Batteries, Electrocatalysis

Correction to: *Nature Communications* 10.1038/s41467-020-15416-4, published online 27 March 2020

During the plotting, an error was inadvertently introduced in the Supplementary Figures 5 and 32 C,d of the original version of this article. The figures are now corrected in both HTML and pdf versions.

## Supplementary information

Updated Supplementary Information

