# Supplementary material for: Atomically dispersed cobalt catalyst anchored on nitrogen-doped carbon nanosheets for lithium-oxygen batteries
Source: Nat Commun. 2020 Mar 27;11:1576. doi: 10.1038/s41467-020-15416-4 (PMC7101366; doi:10.1038/s41467-020-15416-4)
Supplement: Supplementary file 1 — Supplementary Information [file 41467_2020_15416_MOESM1_ESM.pdf]

Supporting Information

Atomically Dispersed Cobalt Anchored Zn-Hexamine Frameworks-Derived Nitrogen-Rich Carbon Nanosheets as Efficient Catalyst for Lithium-Oxygen Batteries

Wang et al

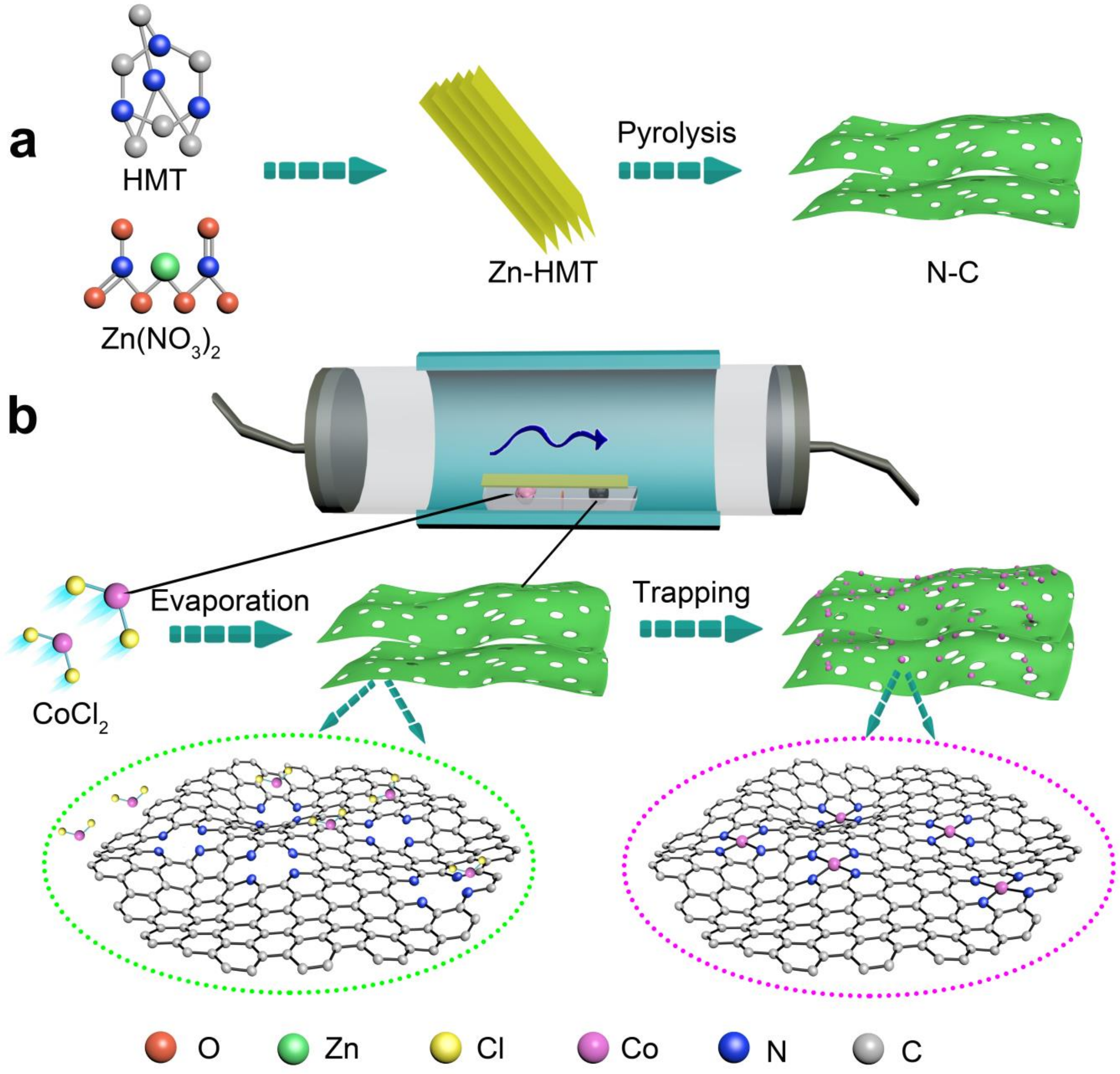

Supplementary Figure 1. Schematic diagram of preparation for Co-SAs/N-C, Co-NPs/N-C and N-C catalysts.

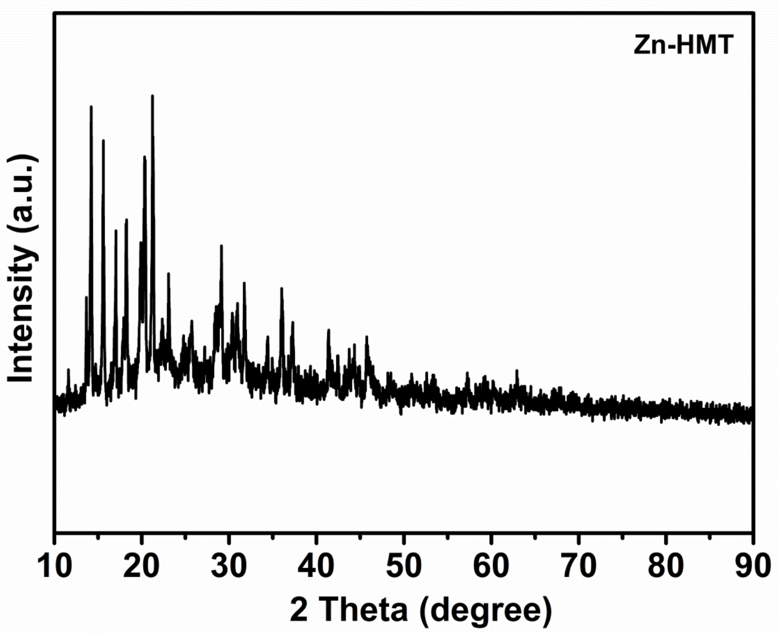

Supplementary Figure 2. XRD pattern of the as prepared Zn-HMT.

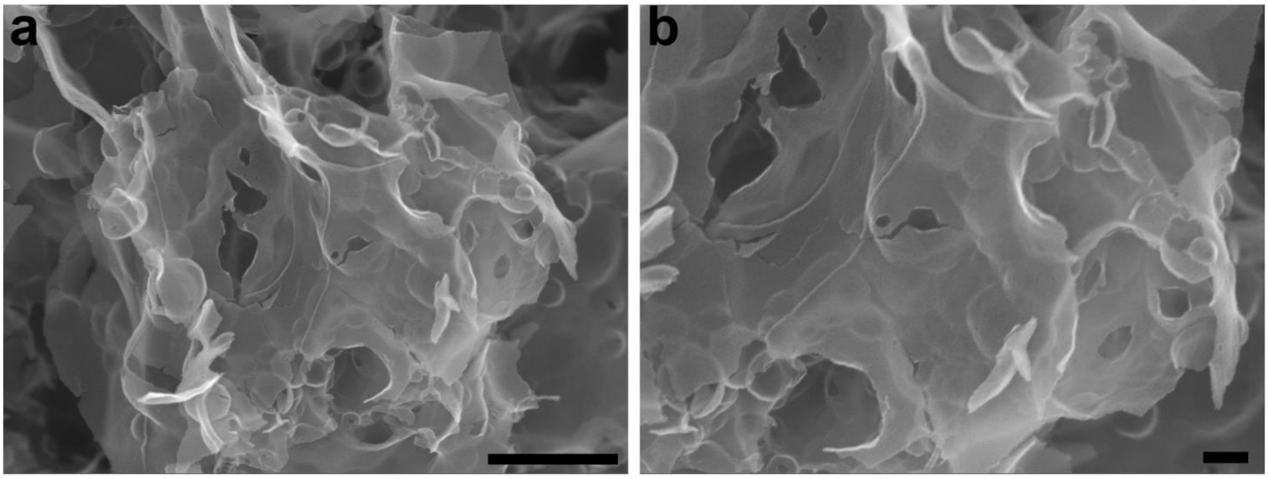

Supplementary Figure 3. SEM images of N-C at different magnifications. **a** Scale bar, 500 nm. **b** Scale bar, 200 nm.

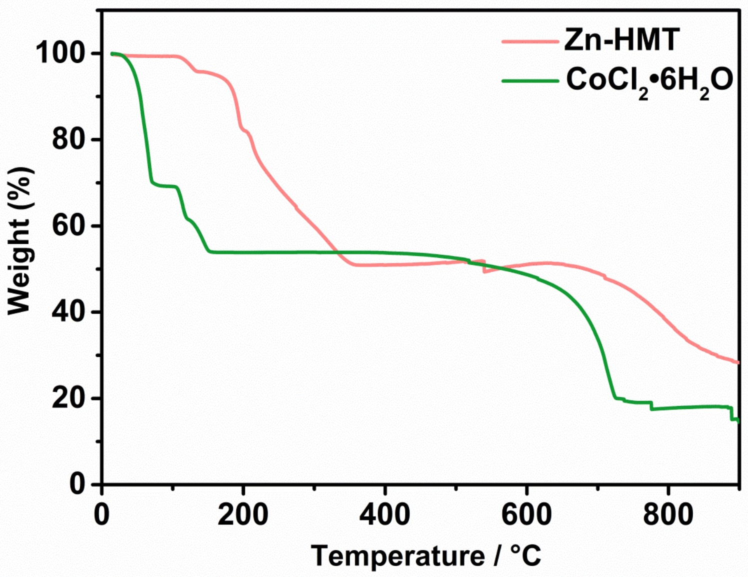

Supplementary Figure 4. TG profiles of Zn-HMT and $CoCl_2{\bullet}6H_2O$ under N_2 atmosphere.

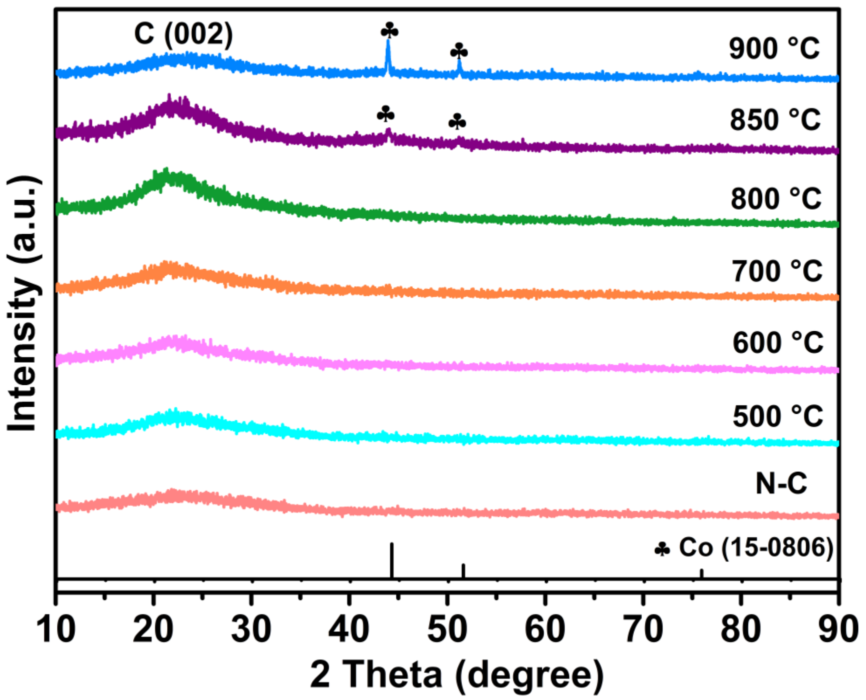

Supplementary Figure 5. XRD patterns of N-C and samples derived from different calcination temperatures.

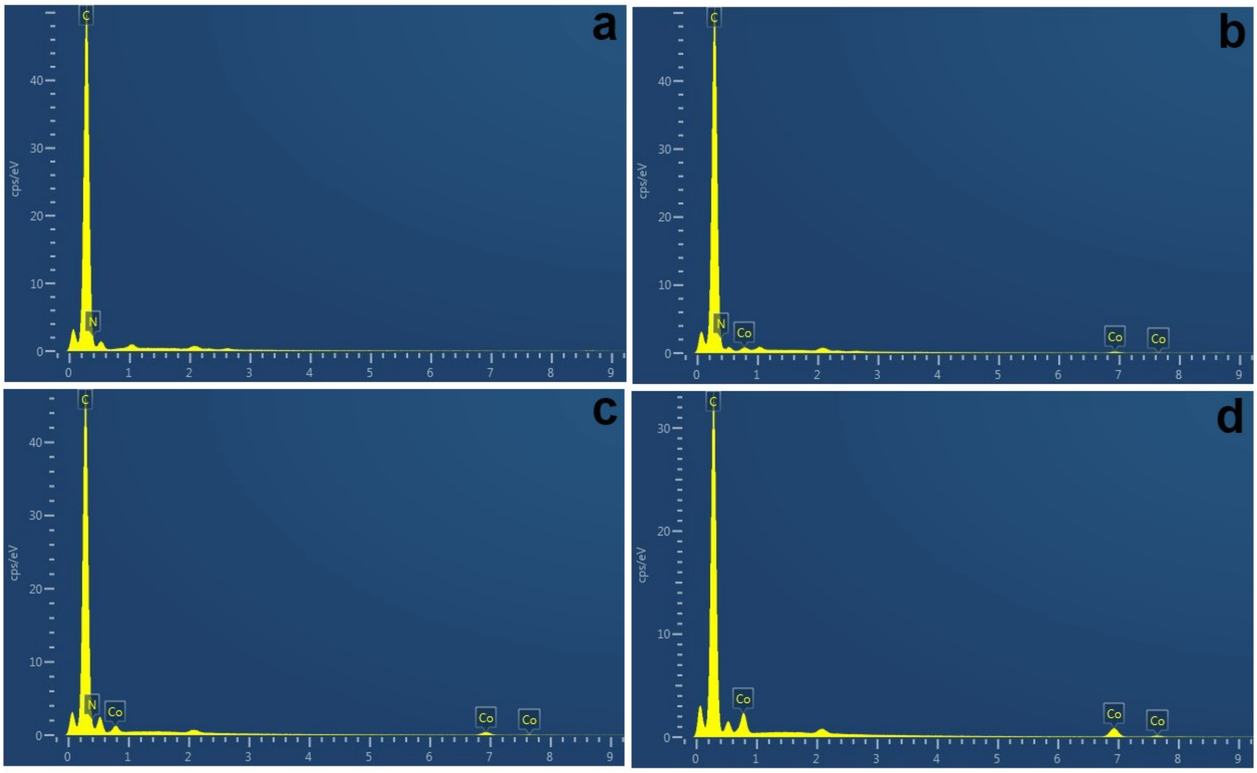

Supplementary Figure 6. EDS patterns of the samples derived from different calcination temperatures. **a** 600 ℃. **b** 700 ℃. **c** 800 ℃. **d** 900 ℃.

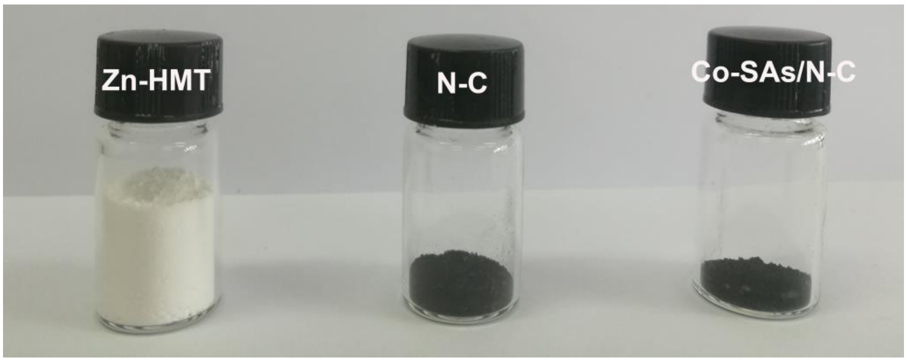

Supplementary Figure 7. The color evolution of Zn-HMT, N-C and Co-SAs/N-C.

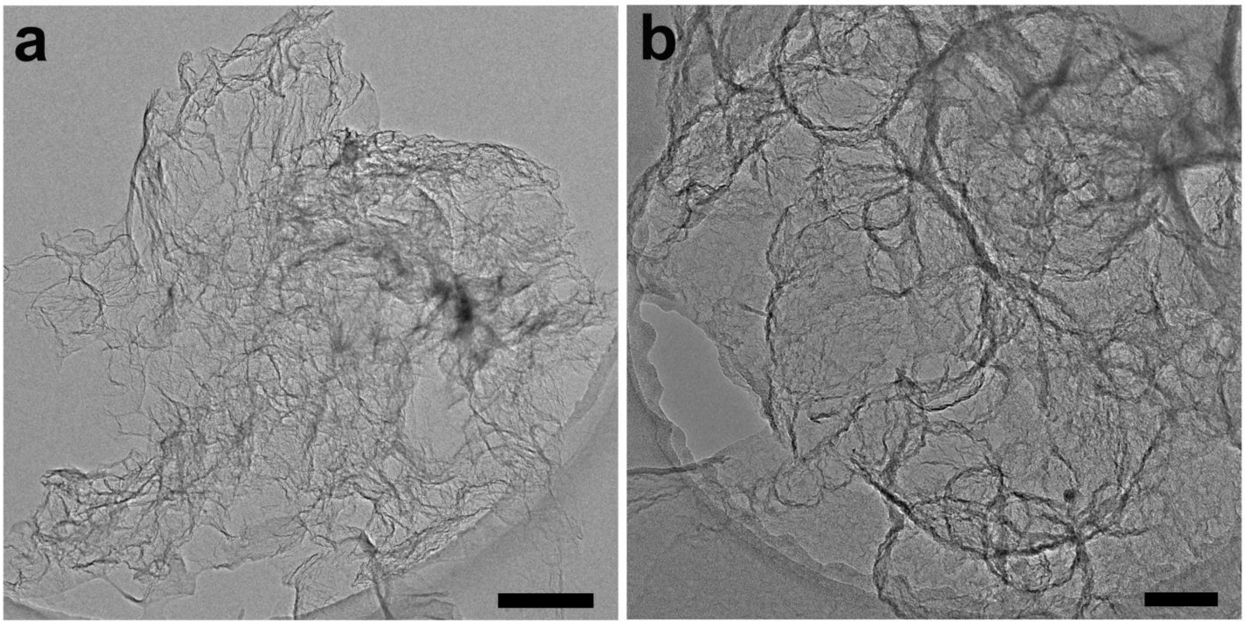

Supplementary Figure 8. TEM images of Co-SAs/N-C at different magnifications. **a** Scale bar, 500 nm. **b** Scale bar, 100 nm.

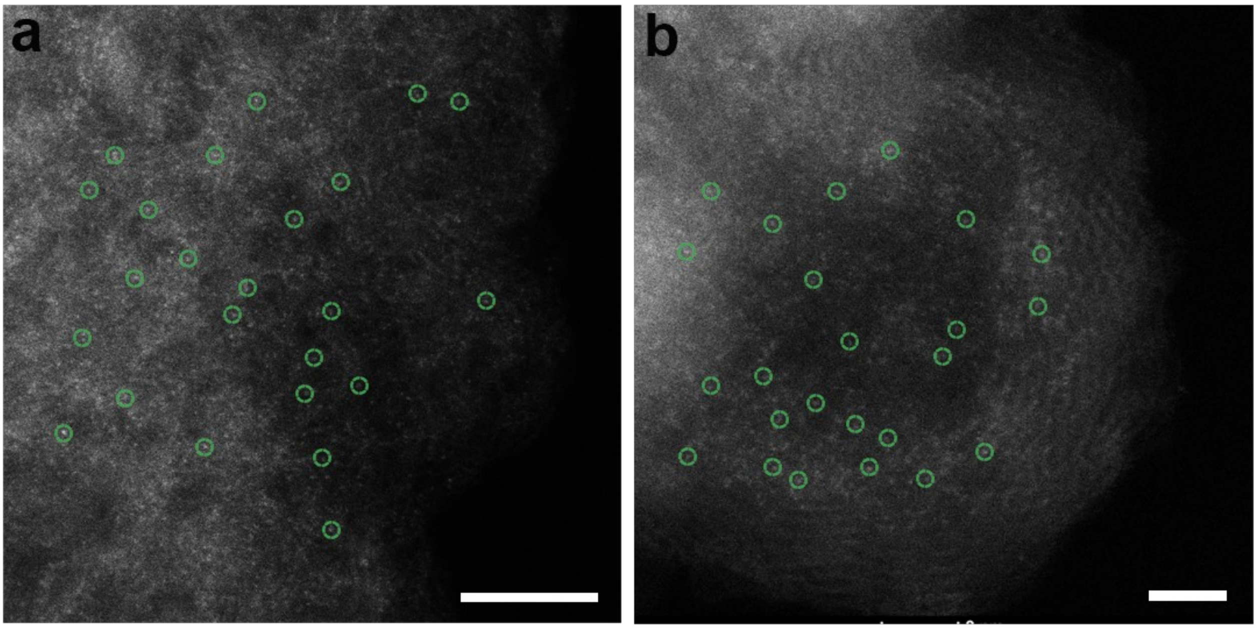

Supplementary Figure 9. HAADF-STEM images of Co-SAs/N-C. **a** Scale bar, 5 nm. **b** Scale bar, 2 nm.

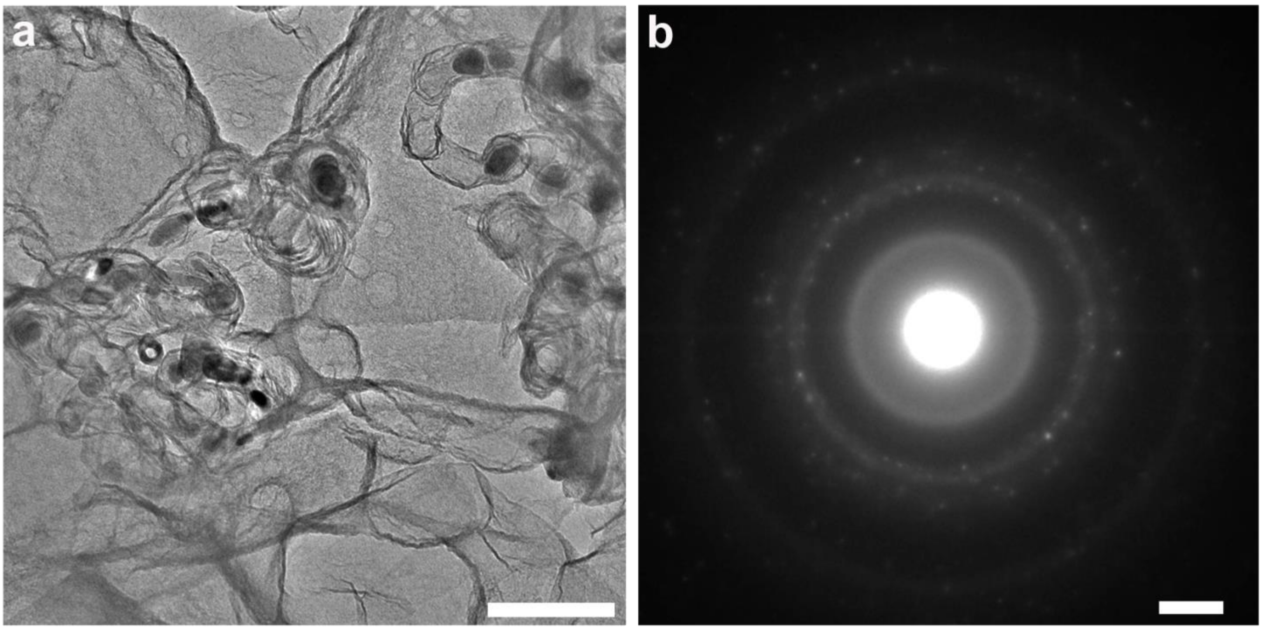

Supplementary Figure 10. TEM and SAED patterns of Co-NPs/N-C. **a** TEM image. Scale bar, 100 nm. **b** SAED pattern. Scale bar, 2 1/nm.

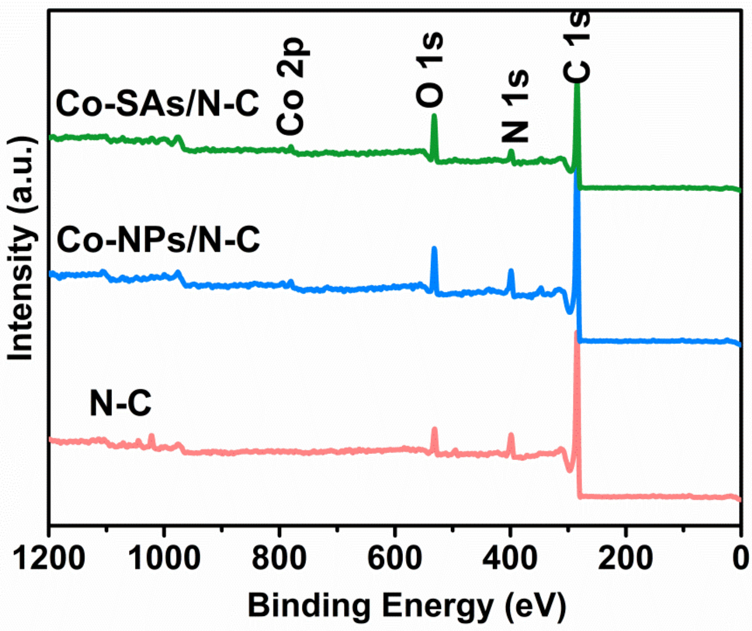

Supplementary Figure 11. XPS survey spectra of Co-SAs/N-C, Co-NPs/N-C and N-C.

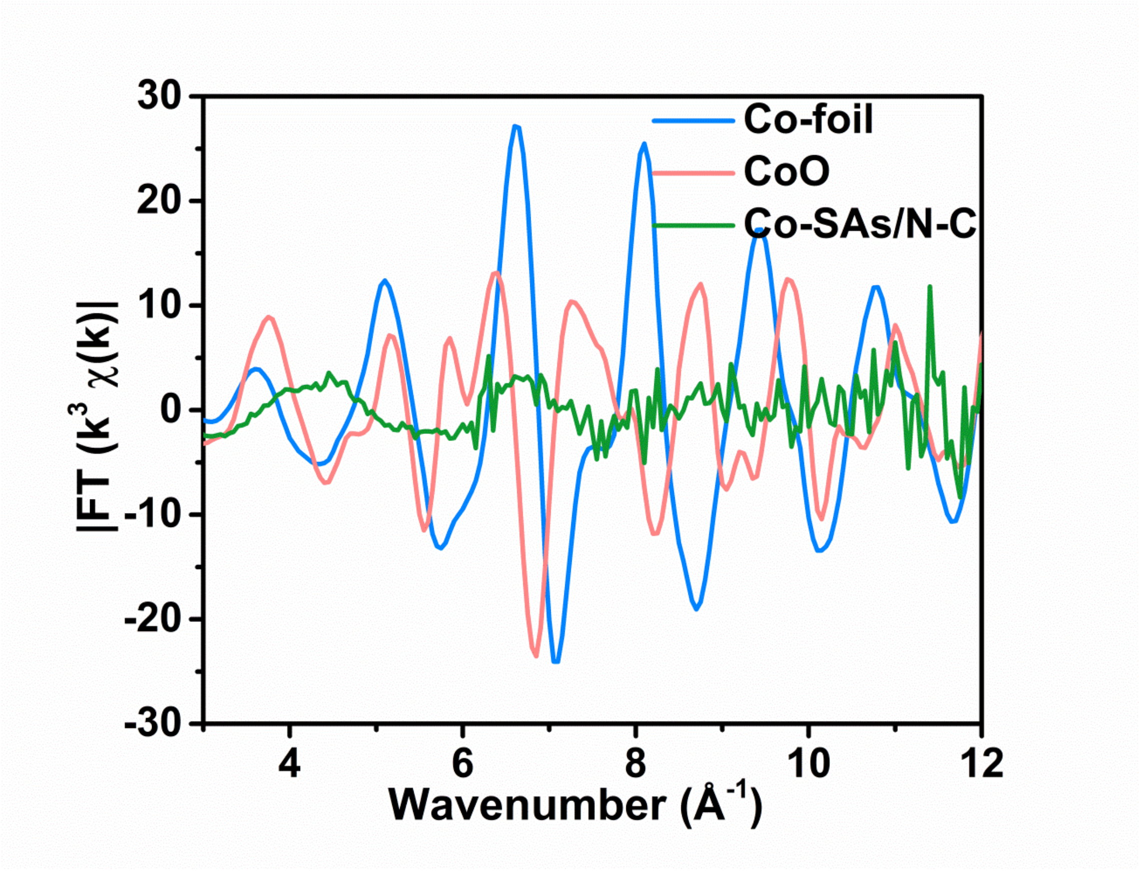

Supplementary Figure 12. EXAFS in K space of Co-SAs/NC, CoO samples and Co foil.

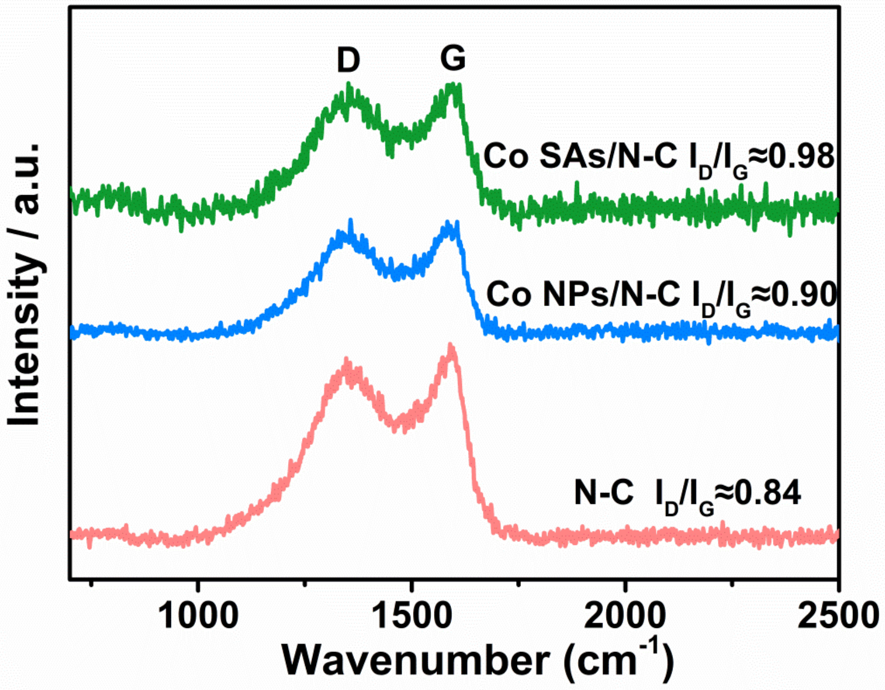

Supplementary Figure 13. Raman spectra of Co-SAs/N-C, Co-NPs/N-C and N-C.

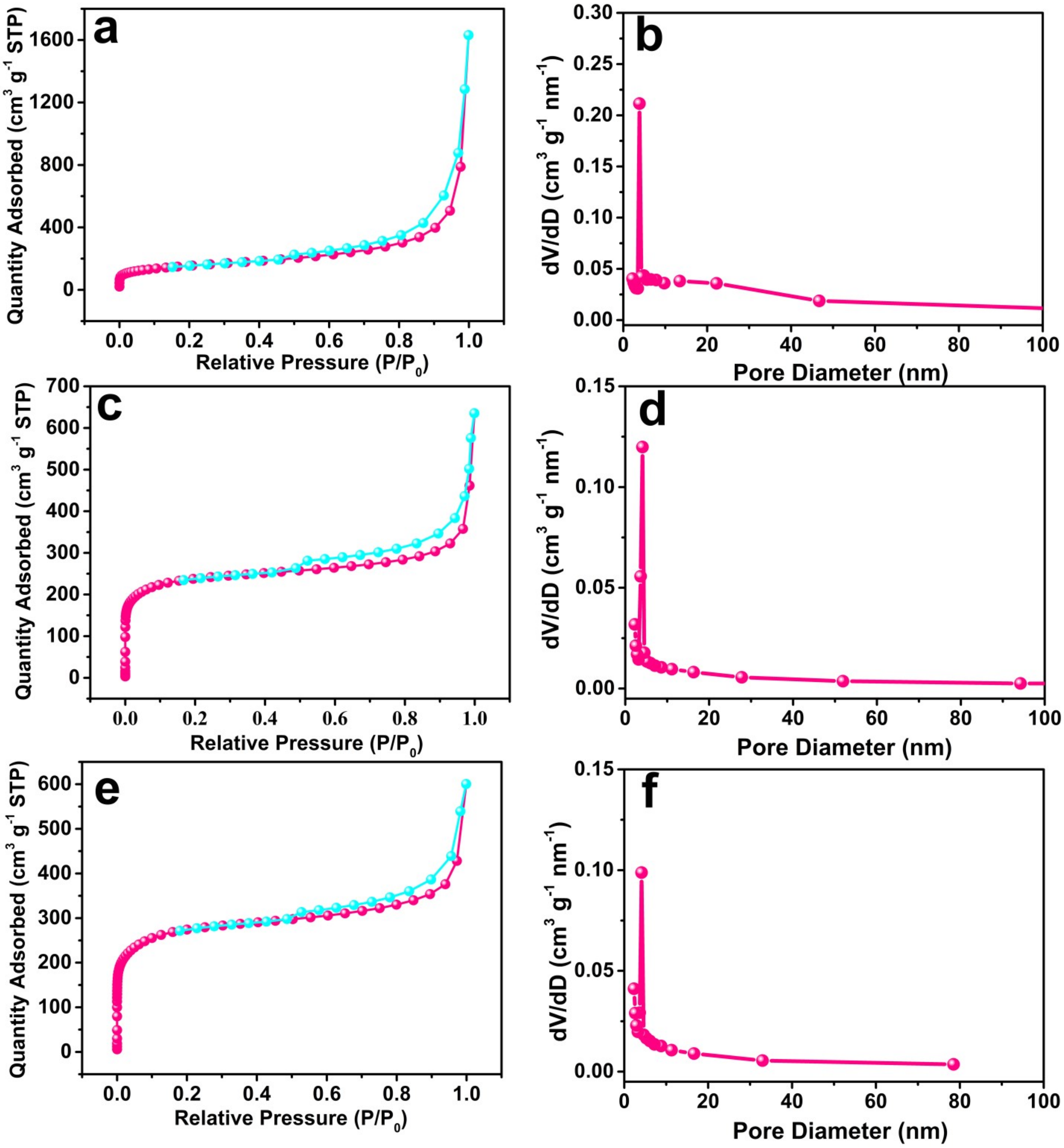

Supplementary Figure 14. Adsorption–desorption isotherms pore size distribution of Co-SAs/N-C, Co-NPs/N-C and N-C. **a, c, e** N_2 adsorption–desorption isotherms. **b, d, f** pore size distribution of Co-SAs/N-C, Co-NPs/N-C and N-C.

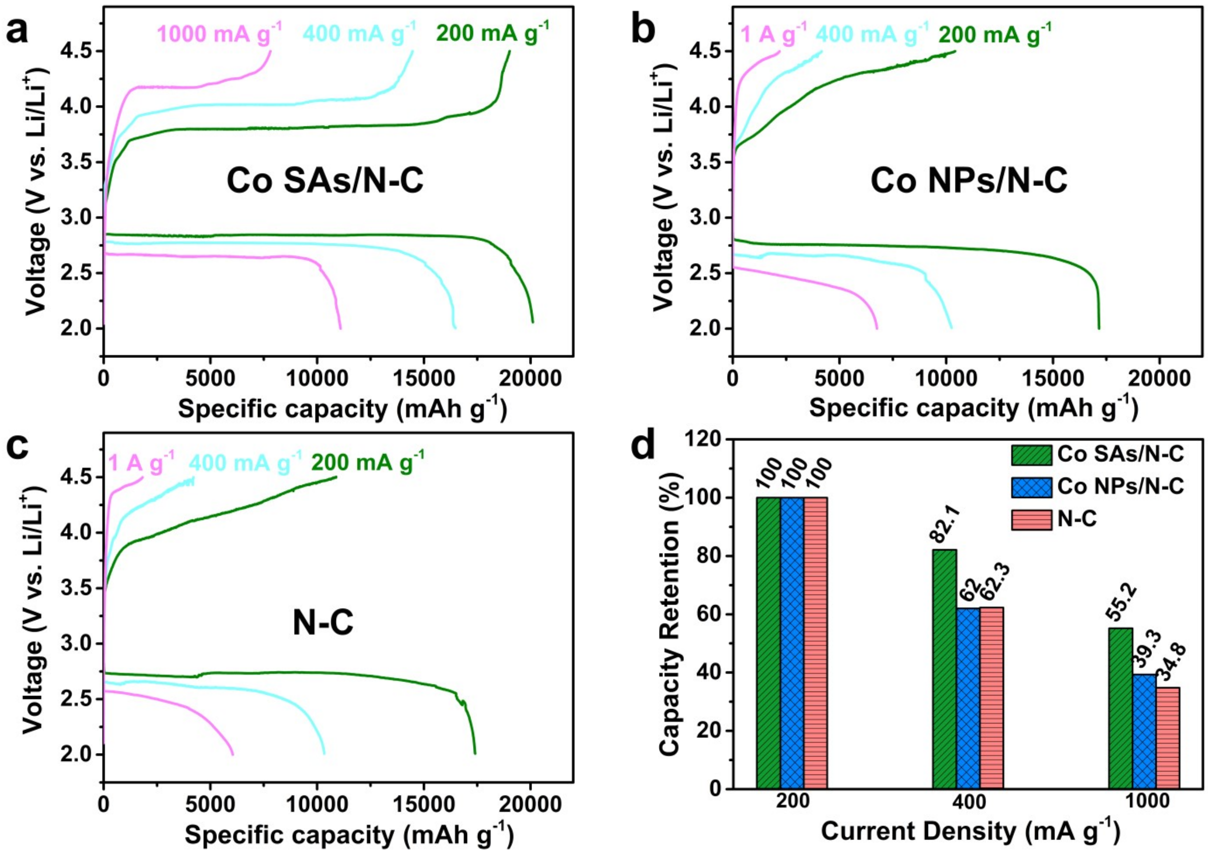

Supplementary Figure 15. The discharge-charge curves and capacity retentions of Co-SAs/N-C, Co-NPs/N-C and N-C. **a-c** The discharge-charge curve at different current densities. **d** The capacity retentions of the three electrodes.

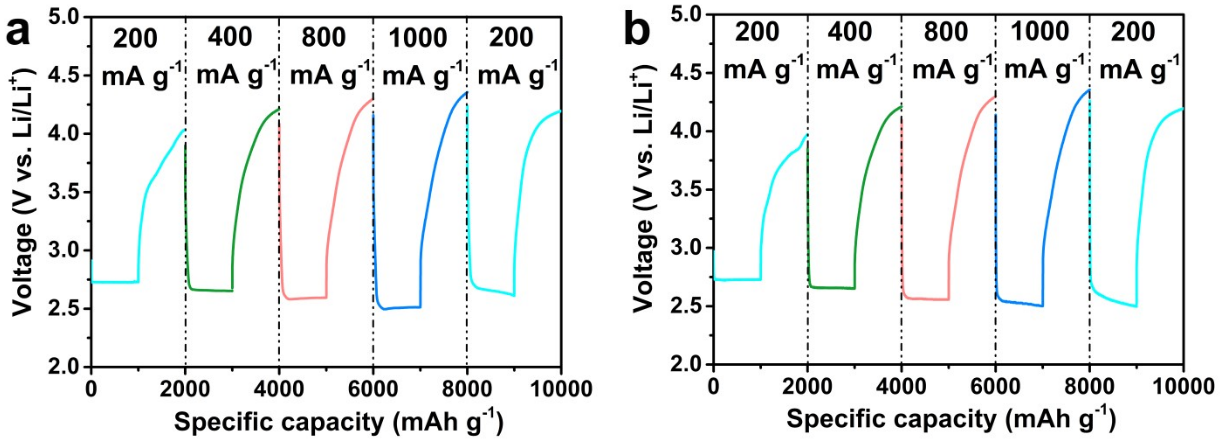

Supplementary Figure 16. The discharge-charge profiles at different current densities. **a, b** The discharge-charge profiles of Co-NPs/N-C and N-C based electrodes at various current densities ranging from 0.2 to 1 A g^{-1} with a limited capacity of 1000 mAh g^{-1}.

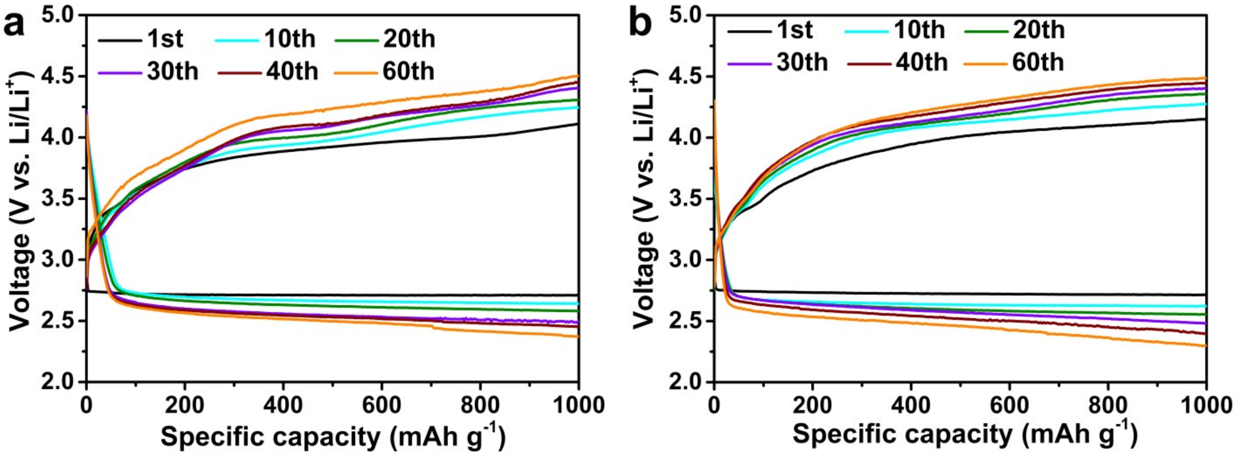

Supplementary Figure 17. Cycling stability of Co-NPs/N-C and N-C. **a, b** The discharge-charge profiles of Co-NPs/N-C and N-C based electrodes with different cycles at 400 mA g^{-1}.

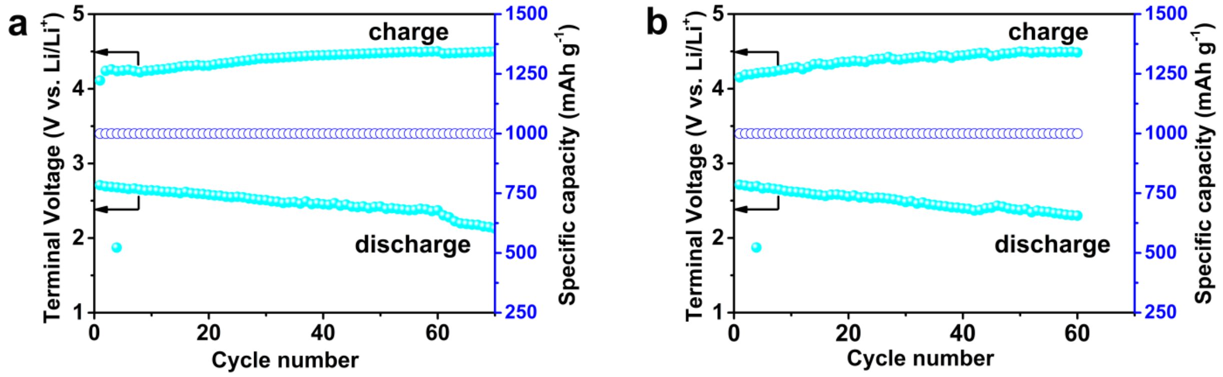

Supplementary Figure 18. Terminal discharge-charge voltages of Co-NPs/N-C and N-C during cycles. **a, b** Cycling stability and terminal discharge-charge voltages of Co-NPs/N-C and N-C electrodes at 400 mA g^{-1} with a limited capacity of 1000 mAh g^{-1}.

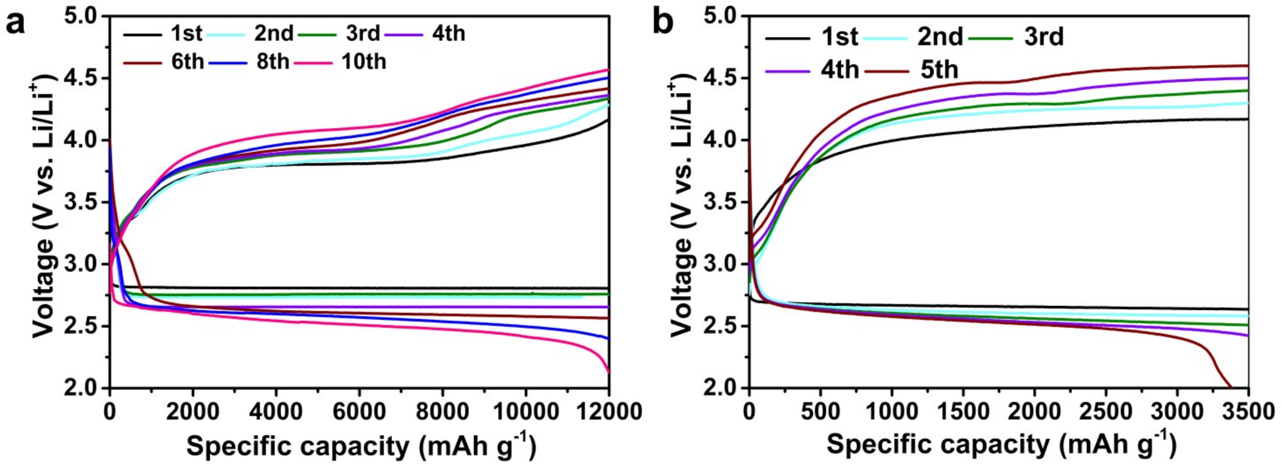

Supplementary Figure 19. The deeper discharge-charge profiles of Co-SAs/N-C and N-C. **a** The discharge-charge profiles of Co-SAs/N-C with different cycles with a cut-off capacity of 12000 mAh g^{-1} at 400 mA g^{-1}, **b** N-C based electrodes with different cycles with a cut-off capacity of 3500 mAh g^{-1} at 400 mA g^{-1}.

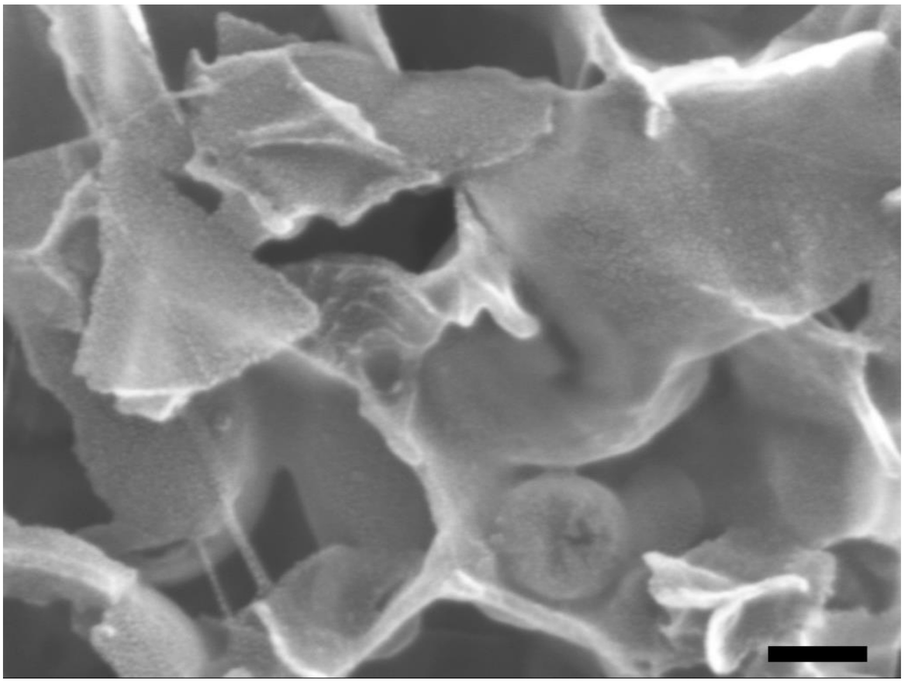

Supplementary Figure 20. EX-situ SEM images of discharged Co-SAs/N-C electrode during the first cycle. Scale bar, 200 nm.

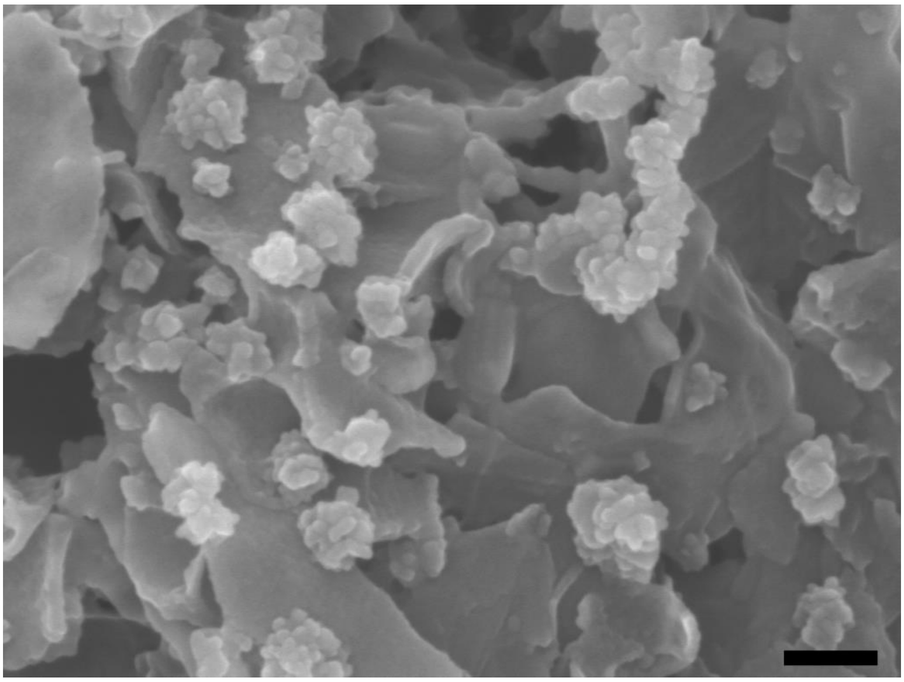

Supplementary Figure 21. EX-situ SEM images of discharged Co-NPs/N-C electrode during the first cycle. Scale bar, 200 nm.

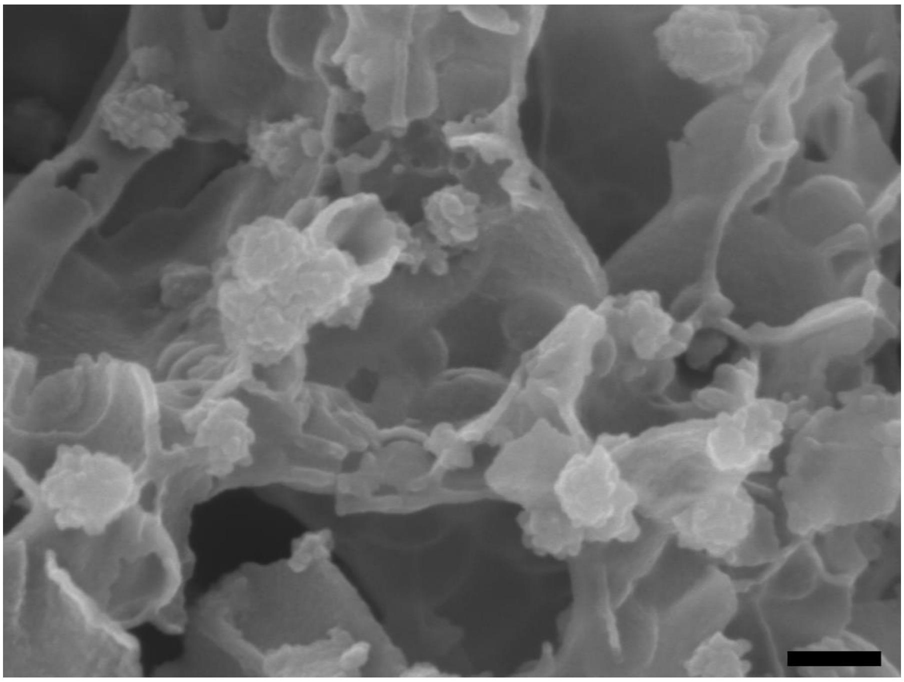

Supplementary Figure 22. EX-situ SEM images of discharged N-C electrode during the first cycle. Scale bar, 200 nm.

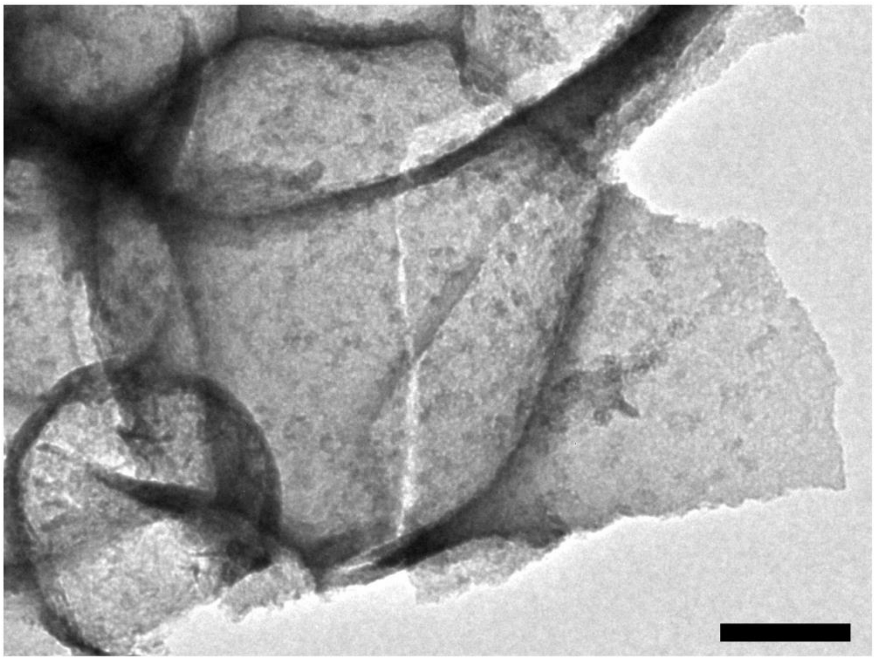

Supplementary Figure 23. EX-situ TEM images of discharged Co-SAs/N-C electrode. Scale bar, 200 nm.

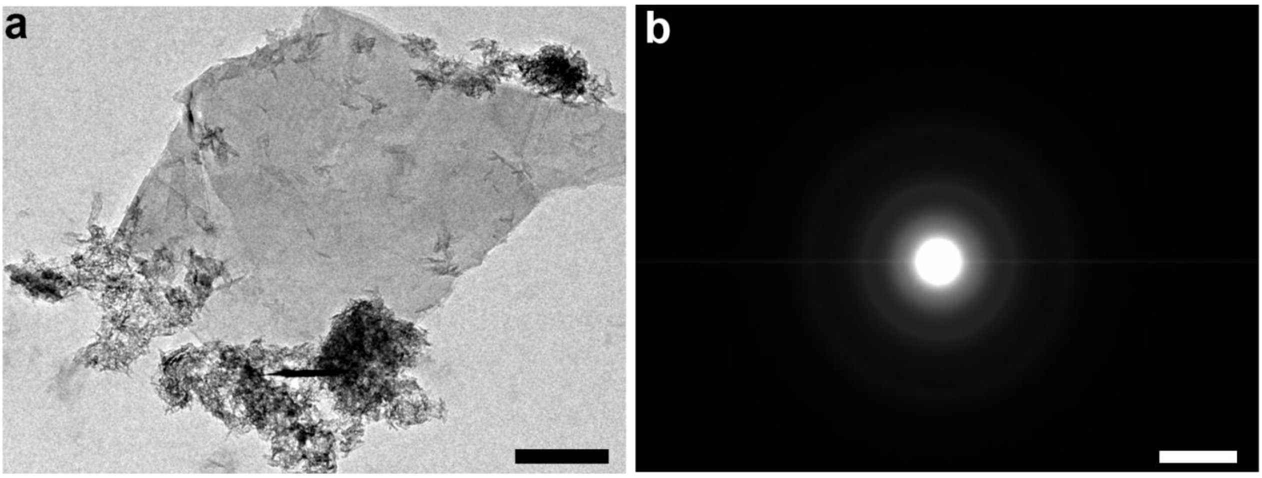

Supplementary Figure 24. EX-situ TEM image and and SAED images of discharged N-C. **a** EX-situ TEM image. Scale bar, 200 nm. **b** EX-situ SAED image. Scale bar, 5 1/nm.

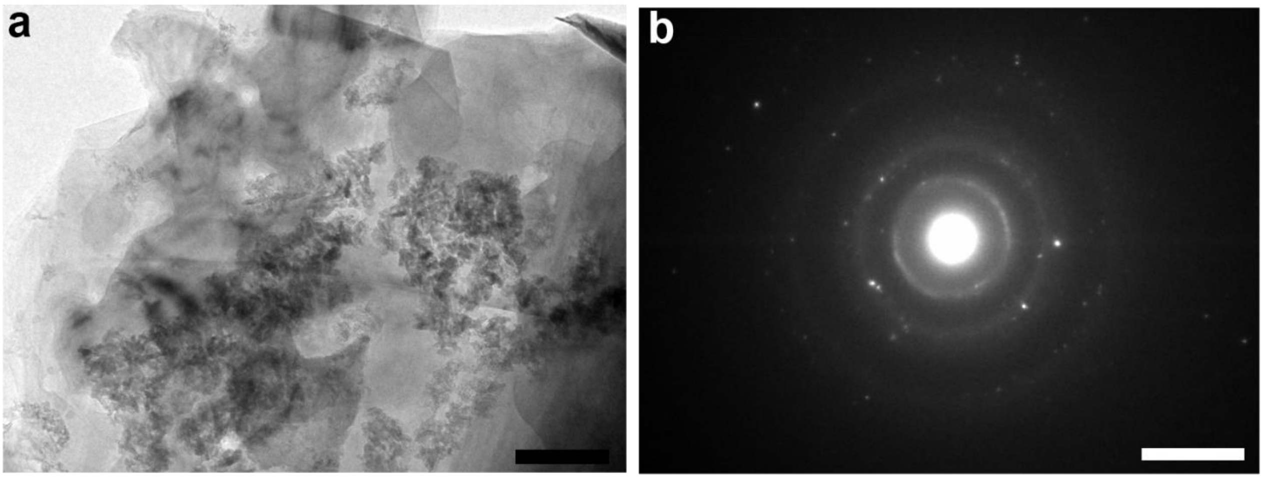

Supplementary Figure 25. EX-situ TEM image and and SAED images of discharged Co-NPs/N-C. **a** EX-situ TEM image. Scale bar, 200 nm. **b** EX-situ SAED image. Scale bar, 5 1/nm.

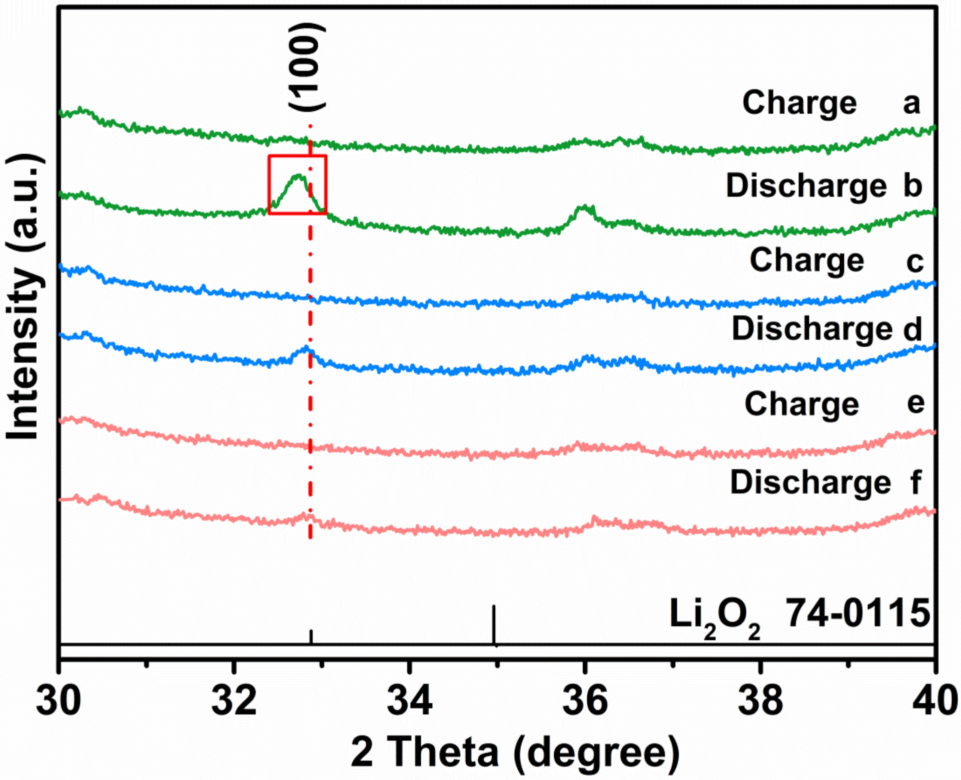

Supplementary Figure 26. EX situ XRD patterns of recharged and discharged electrodes. **a, b** Co-SAs/N-C. **c, d** Co-NPs/N-C. **e, f** N-C.

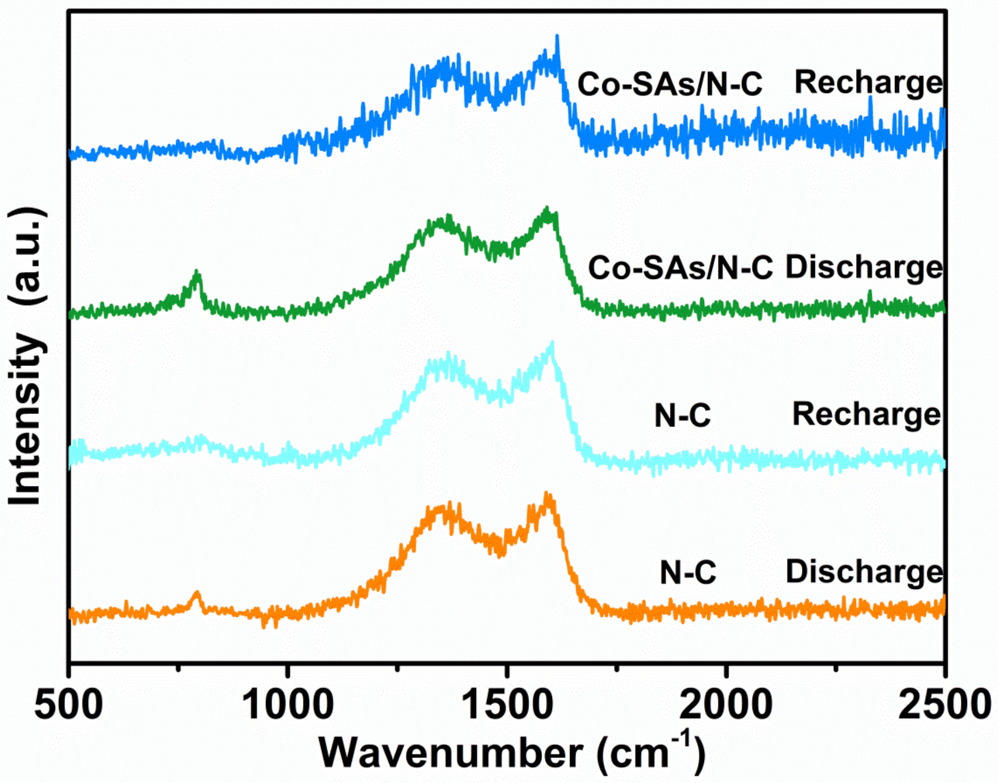

Supplementary Figure 27. Raman spectra of the discharged/charged electrodes for Co-SAs/N-C and N-C at the1st full cycle.

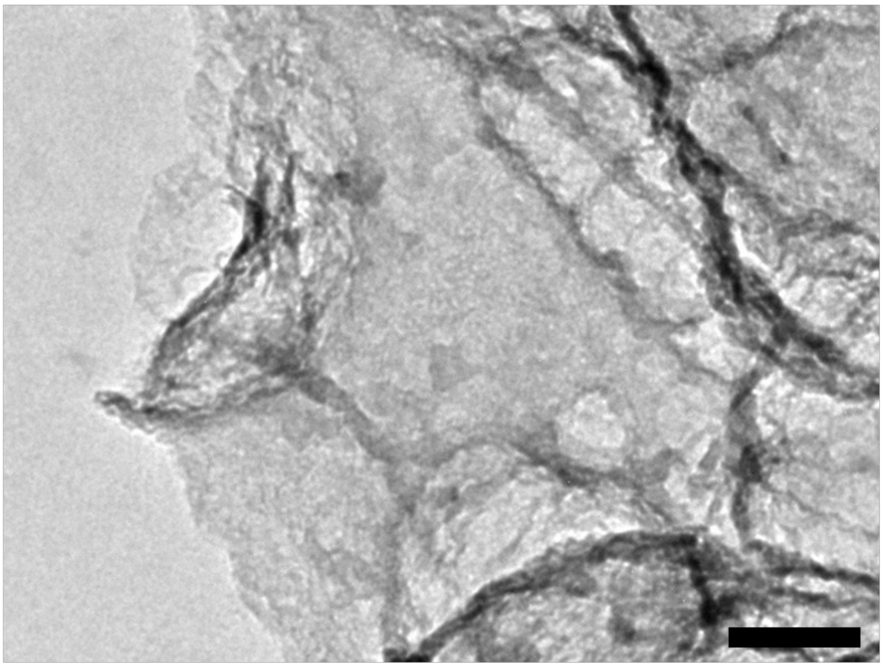

Supplementary Figure 28. EX-situ TEM images of recharged Co-SAs/N-C catalyst. Scale bar, 200 nm.

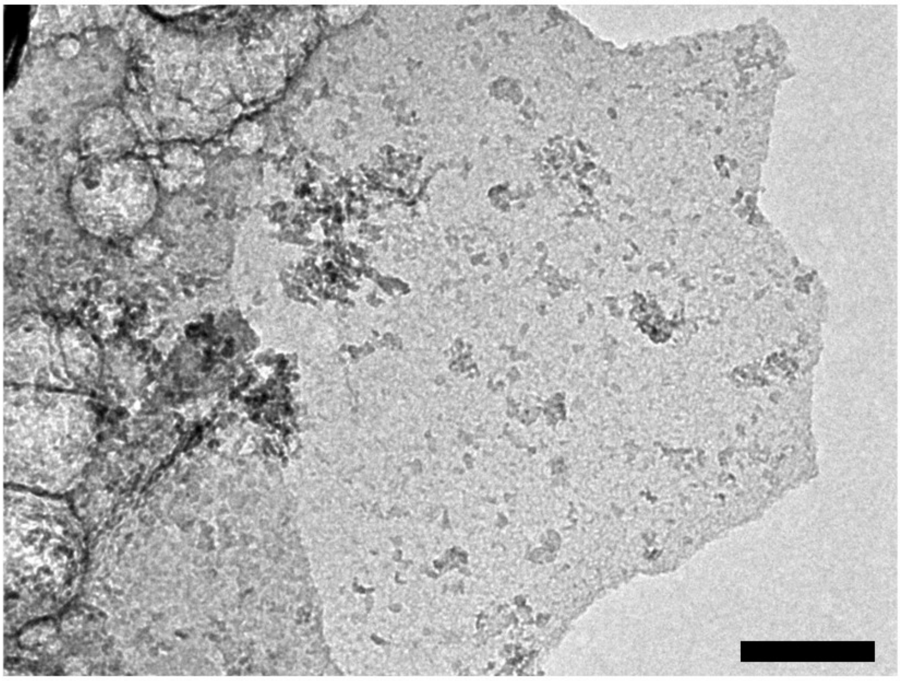

Supplementary Figure 29. EX-situ TEM images of recharged N-C catalyst. Scale bar, 200 nm.

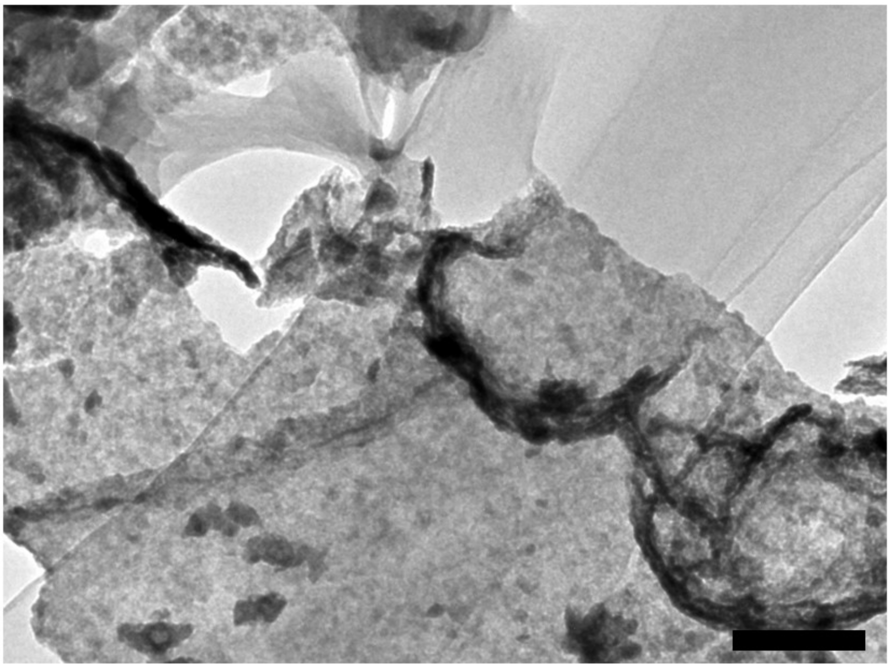

Supplementary Figure 30. EX-situ TEM images of recharged Co-NPs/N-C catalyst. Scale bar, 200 nm.

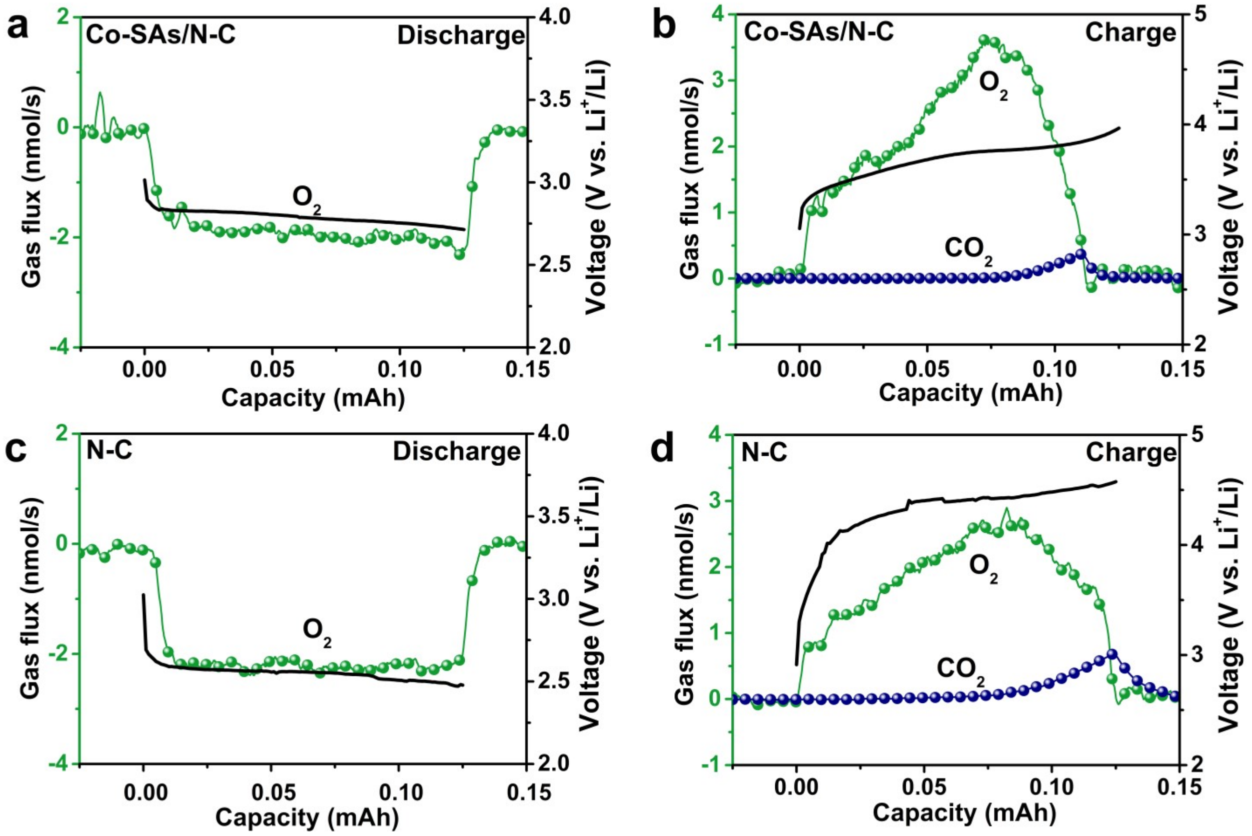

Supplementary Figure 31. Differential electrochemical mass spectrometry (DEMS) analysis. **a, b** DEMS analysis of the evolved gases during the discharge and charge of a Li-O_2 cell with Co-SAs/N-C cathode. **c,d** DEMS analysis of the evolved gases during the c) discharge and d) charge of a Li-O_2 cell with N-C cathode. The solid black lines indicate the potential, while the green and blue solid dots denote the O_2 and CO_2 evolution profiles, respectively.

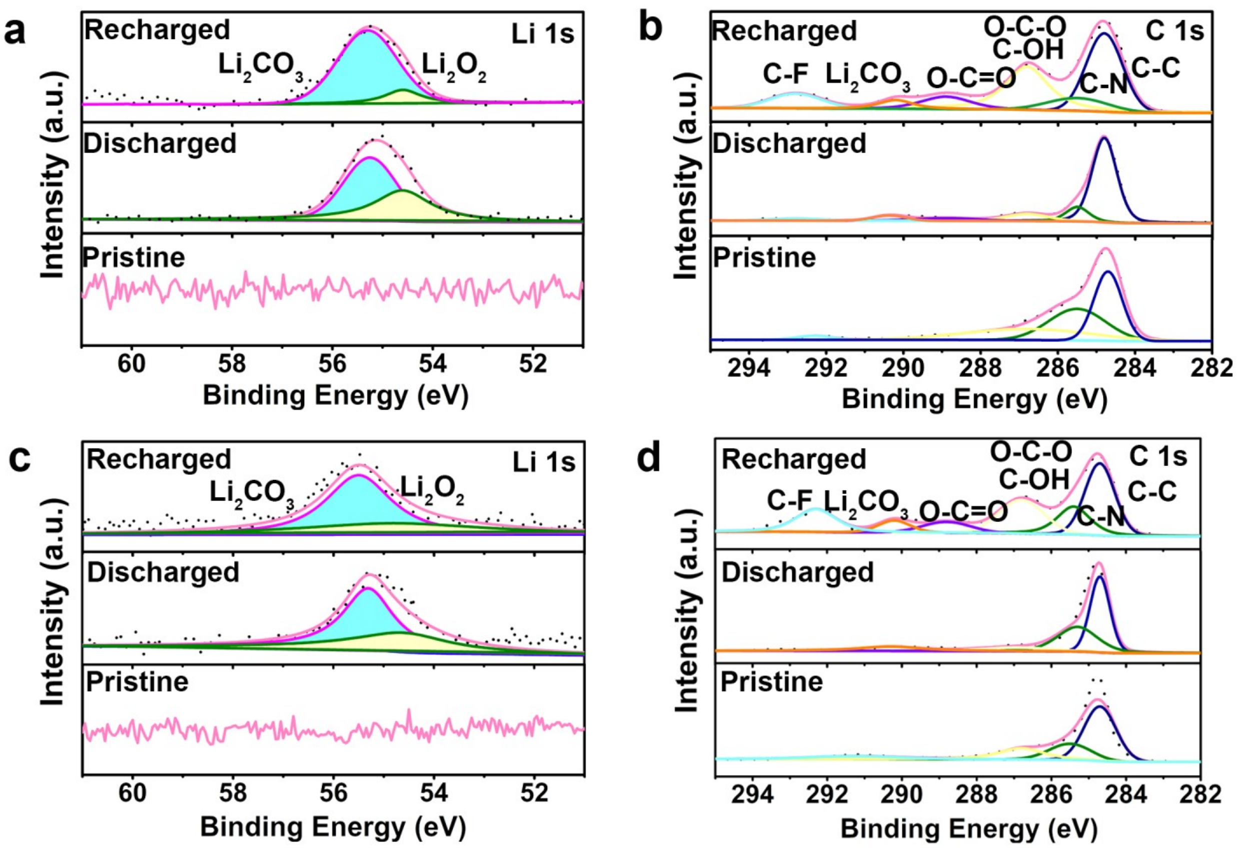

Supplementary Figure 32. Ex-situ XPS analysis. **a, b** Ex-situ XPS spectra of pristine, discharged and recharged Co-NPs/N-C electrode in Li 1s and C 1s regions. **c, d** Ex-situ XPS spectra of pristine, discharged and recharged N-C electrode in Li 1s and C 1s regions.

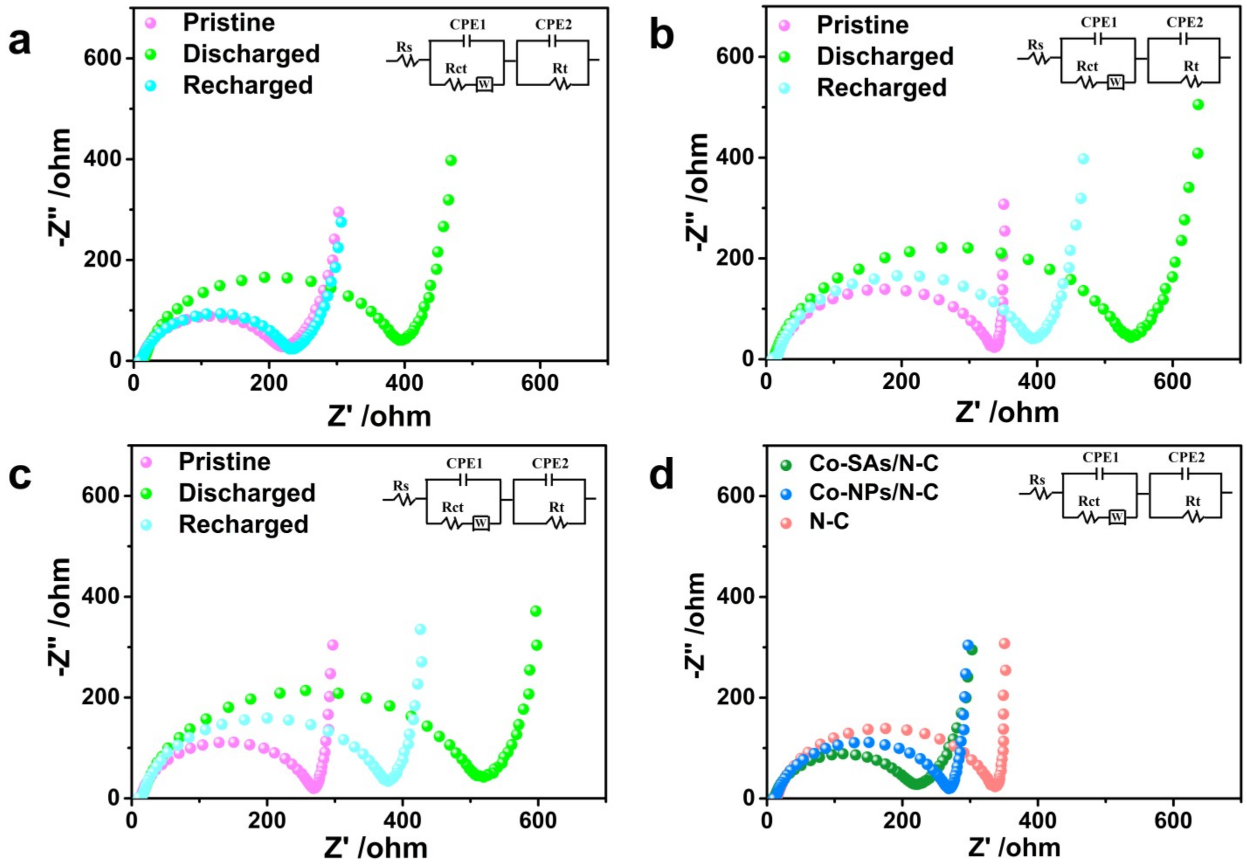

Supplementary Figure 33. Nyquist plots analysis. **a-c** Nyquist plots of Co-SAs/N-C, Co-NPs/N-C and N-C based electrodes at different discharge/charge stages. **d** Nyquist plots of the fresh Co-SAs/N-C, Co-NPs/N-C and N-C electrodes.

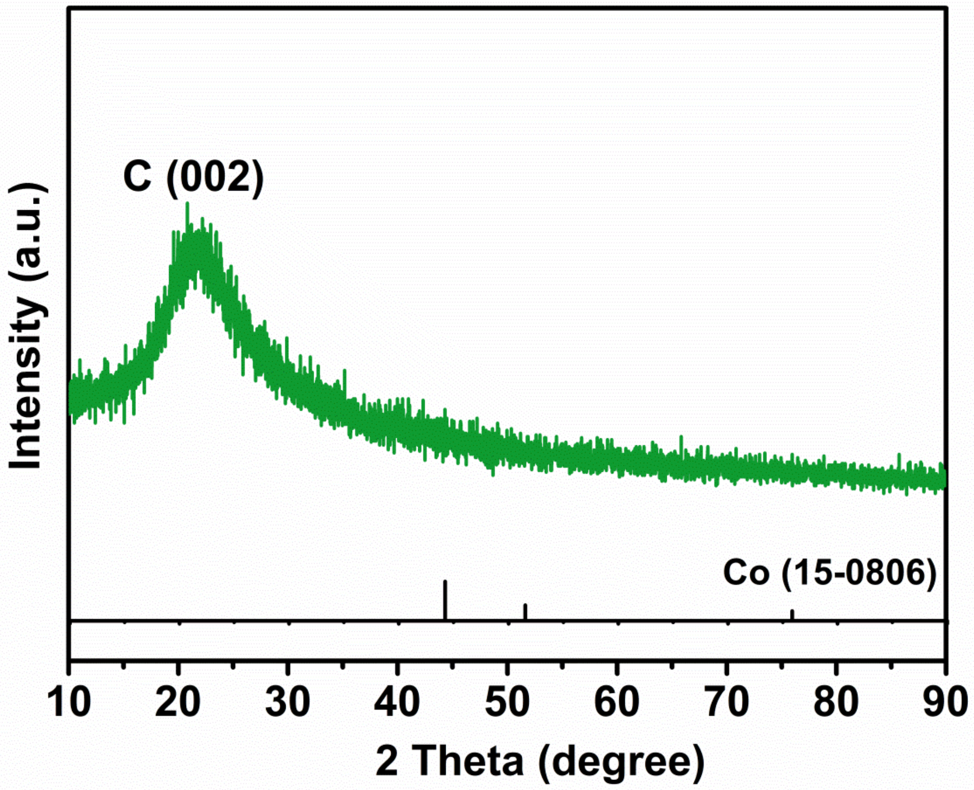

Supplementary Figure 34. XRD pattern of Co-SAs/N-C catalyst powders scratched from the electrode after the 200th cycle.

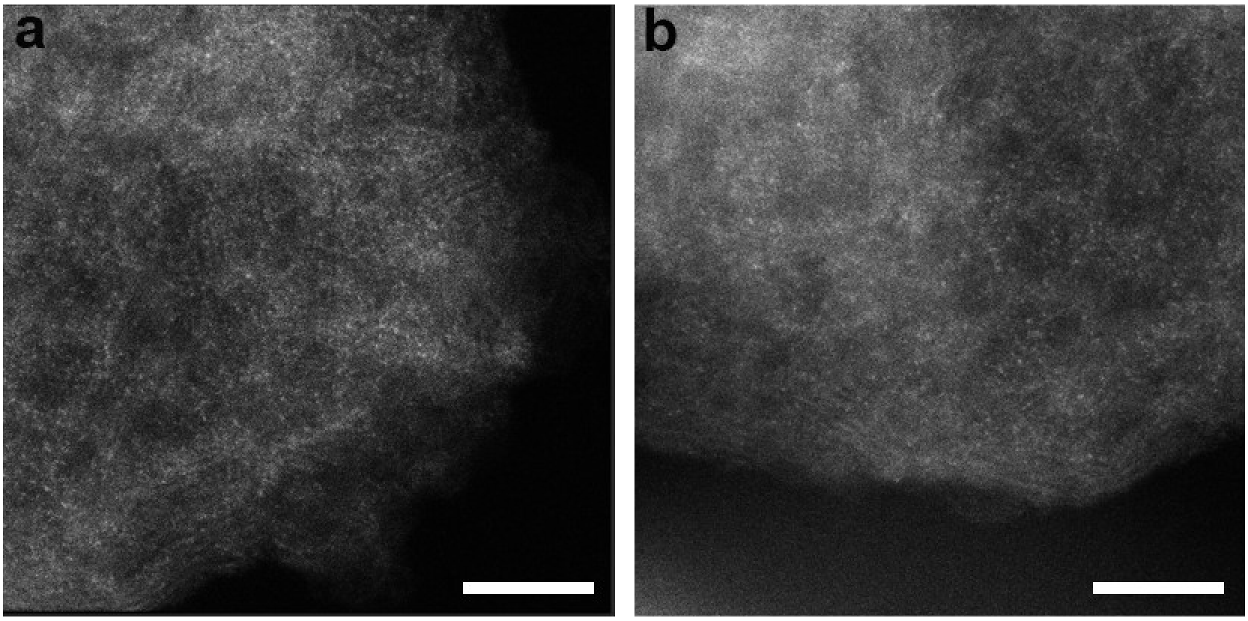

Supplementary Figure 35. HAADF-STEM images. **a, b** HAADF-STEM images of Co-SAs/N-C catalyst scratched from the electrode at different areas after the 200th cycle. Scale bar, 5 nm.

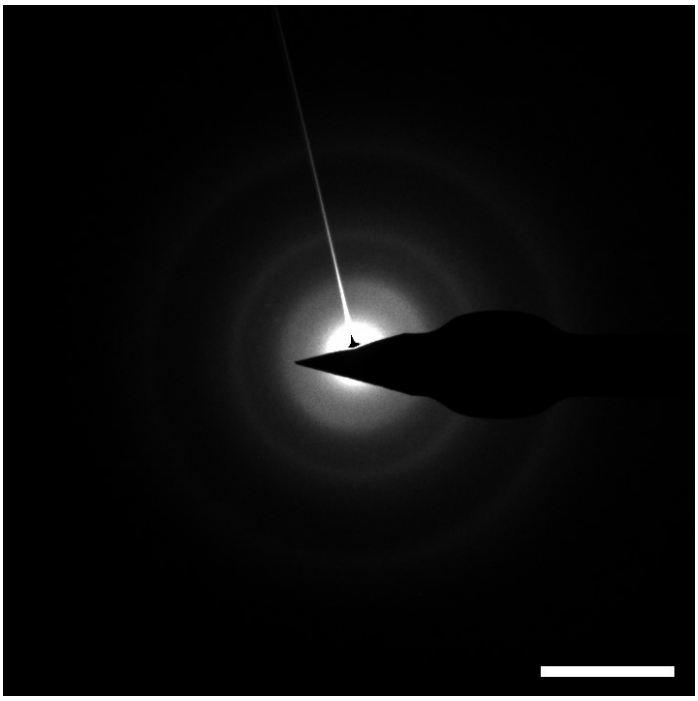

Supplementary Figure 36. SAED pattern of Co-SAs/N-C catalyst scratched from the electrode after the 200th cycle. Scale bar, 5 1/ nm.

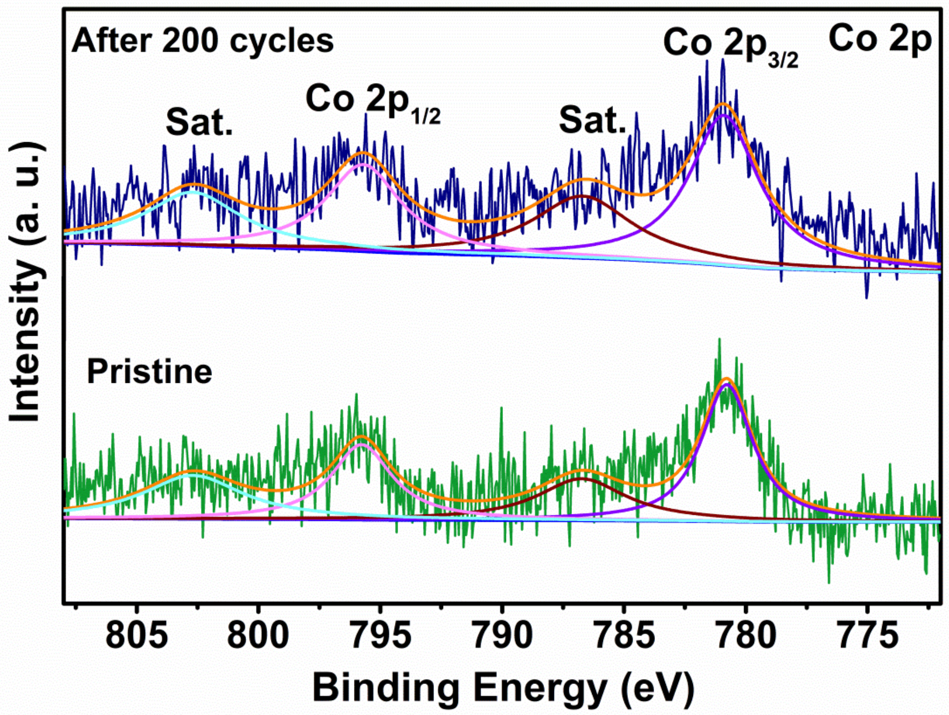

Supplementary Figure 37. XPS patterns of the pristine Co-SAs/N-C and the Co-SAs/N-C catalyst scratched from the electrode after the 200th cycle.

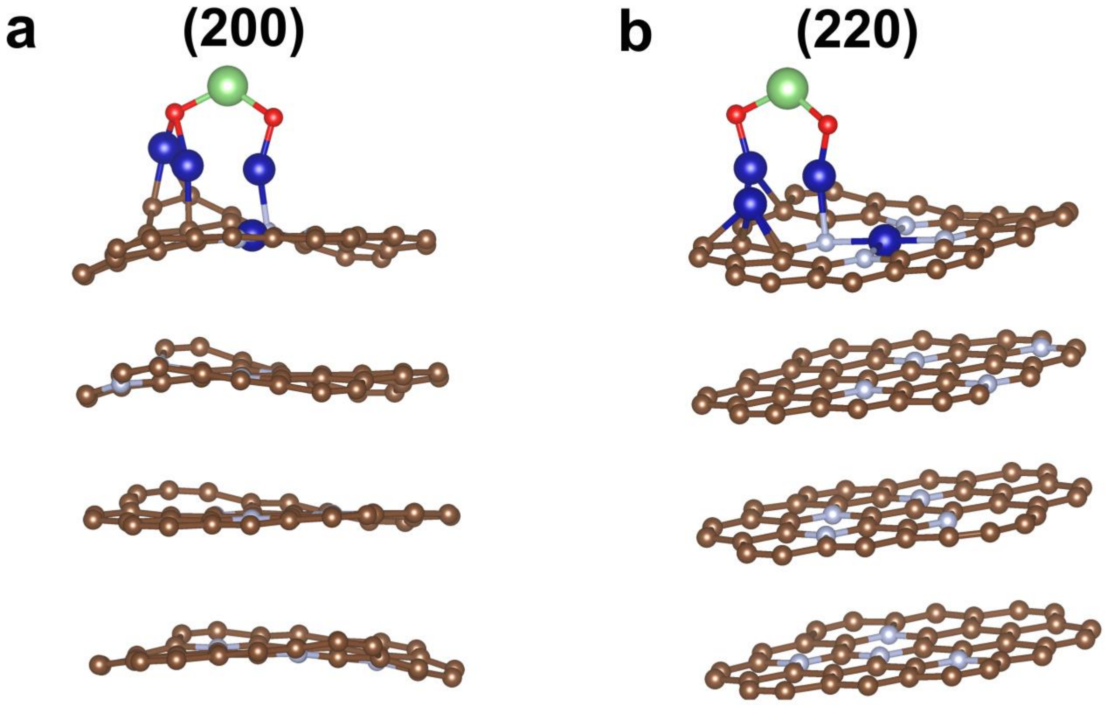

Supplementary Figure 38. The optimized structures of LiO_2 adsorbed on different planes. **a** (200) plane of Co nanoparticle in Co-NPs/N-C. **b** (220) plane of Co nanoparticle in Co-NPs/N-C.

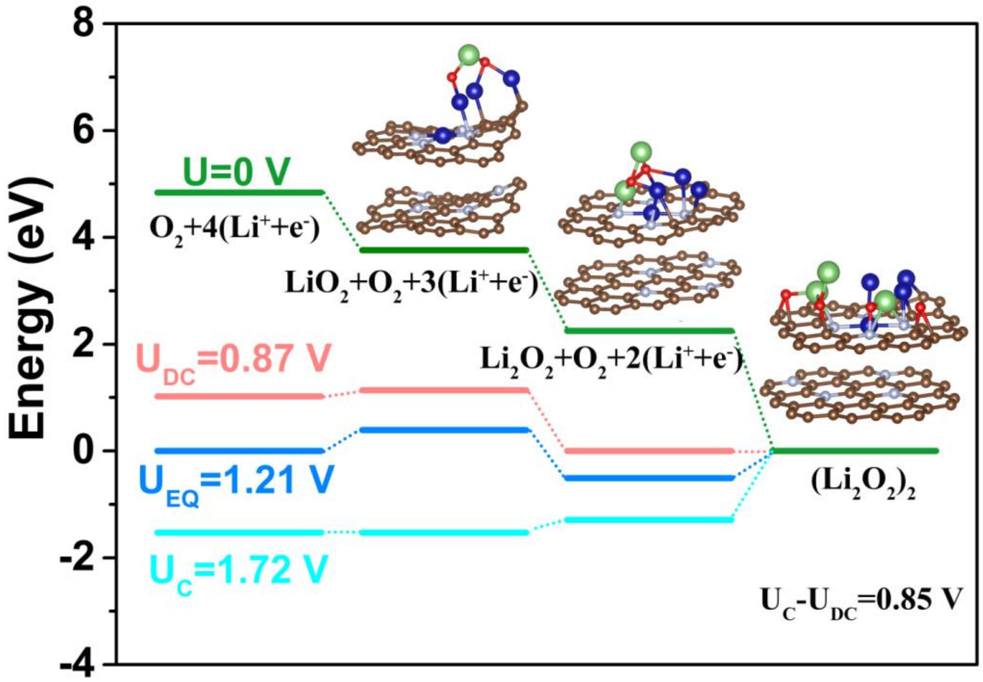

Supplementary Figure 39. Calculated free energy diagrams for the discharge-charge reactions on the active surface of Co-NPs/N-C.

Supplementary Table 1. Elemental compositions of catalysts determined by XPS (C, N, O, Co) and ICP-OES (Co)

Catalysts materials	C (wt. %)	N (wt. %)	O (wt. %)	Co (wt. %) XPS	Co (wt. %) ICP-OES
N-C	85.36	10.95	3.69	/	/
Co-NPs/N-C	78.44	9.91	3.53	8.12	8.07
Co-SAs/N-C	82.37	10.28	4.78	2.57	2.01

Supplementary Table 2. Co K-edge EXAFS curve fitting parameters. (S_0^2=0.90)

Catalyst	Shell	N	R(Å)	σ^2 (×10^{-3}Å^2)	ΔE_0 (eV)	R%
Co-SAs/N-C	Co-N	3.8	1.94	4.0	-2.3	1.0
Co foil	Co-Co	12.0	2.48	6.9	-5.8	0.49
CoO	Co-Co	12.0	3.09	8.6	3.9	0.87
	Co-O	6.0	2.17	7.4	5.3	0.72

N: coordination number;
R: interatomic distance between central atoms and backscatter atoms;
σ^2: Debye-Waller factor to characterize both thermal and structural disorders;
ΔE_0: inner potential shift;
R%: the indicator for the goodness of the fit.
S_0^2: the amplitude reduction factor.
Error bounds (accuracies) are estimated as N, ±0.1; R, ±0.02; σ^2, ±1.60; ΔE_0, ±0.50.

Supplementary Table 3. Comparison of electrochemical performance between this work and some reported doped C-based and noble metal-based cathodes for Li-O_2 batteries

Catalysts materials	Overpotential (V)	Current density /cut-off capacity (mA g^{-1}/mAh g^{-1})	Cycle number (cycles)	Reference
N-CNT	0.97	500/1000	126	2
Ru/C	0.91	500/2000	125	3
N-Graphene/RuO_2	1.10	400/2000	100	4
Au/CNT	1.04	400/500	112	5
Co_9S_8/C foil	0.57	100/500	105	6
MnO_2/RuO_2/Graphene	0.37	100/1000	45	7
Co[Co,Fe]O_4/N-Graphene	/	100/500	110	8
CNT film	1.72	200/1000	262	9
MoO_x/CNT	0.75	200/1000	210	10
IrCo/porous C	0.77	200/1000	200	11
MnO_2/C nanosheet	1.22	200/1000	28	12
N-C/CNT	c.a. 1.20	200/500	75	13
**This work**	**0.4**	**400/1000**	**260**	

Supplementary Note 1. The programmable fabrication process of the catalysts

The programmable fabrication process of the Co-SAs/N-C catalyst is illustrated in **Supplementary Figure 1**. Firstly, Zn-HMT white precipitations are successfully synthesized by mixing hexamine (HMT) with abundant N-donor ligands as nitrogen/carbon source and $Zn(NO_3)_2 \cdot 9H_2O$ in ethanol solution through metal-ligand interaction. Subsequently, the as-fabricated bulk Zn-HMT can be thermally exfoliated into ultrathin N-doped carbon nanosheets (N-C) through a fast pyrolysis process at 900 °C under Ar atmosphere[1]. At last, a gas-migration-trapping procedure is carried out. The Co-SAs/N-C and Co-NPs/N-C catalysts are generated just by tuning the annealing temperatures from 500 to 900 °C according to TG curves in **Supplementary Figure 4**.

Supplementary Note 2. XRD patterns of N-C and samples derived from different calcination temperatures

As shown in the corresponding X-ray diffraction (XRD) patterns (**Supplementary Figure 5**), when the temperatures are controlled below 800 ℃, only a typical diffraction peak indexed to (002) plane of graphitic carbon exists. However, when the temperatures are elevated to 850 and 900 ℃, three distinct reflection peaks at 44.2°, 51.5° and 75.8° gradually appear, implying Co nanoparticles may form (JCPDS Card No. 15-0806).

Supplementary Note 3. EDS patterns of the samples derived from different calcination temperatures

Energy-dispersive X-ray spectroscopy (EDS) spectra of the samples in **Supplementary Figure 6** also identify the component evolution and demonstrate temperature is the crucial parameter in determining whether the isolated Co atoms can be decorated into nitrogenated carbon again. We postulate Co^{2+} ions cannot be bound with N-C at lower temperature (500-600 ℃) and Co nanoparticles inevitably appear at high temperature (850-900 ℃). In this regard, N-C, Co-SAs/N-C (800 ℃), Co-NPs/N-C (900 ℃) species are chosen for the following investigations.

Supplementary Figure 4. Raman spectra of the discharged/charged electrodes for Co-SAs/N-C and N-C at the1st full cycle

As shown in **Supplementary Figure 27**, the intense peaks at ~798 cm^{-1} which are characteristic for Li_2O_2, demonstrate that Li_2O_2 dominates the discharge products. After a following recharge, the Li_2O_2 peak vanishes, further confirming an excellent reversibility associated with the desirable formation and decomposition of Li_2O_2, which is in accordance with the XRD results in **Figure 4e**.

Supplementary References

1. Liu, S., Zhou, J. & Song, H. 2D Zn-hexamine coordination frameworks and their derived N-rich porous carbon nanosheets for ultrafast sodium storage. *Adv. Energy Mater.* 8, 1800569 (2018).
2. Yang, X. Y. et al Blood-capillary-inspired, free-standing, flexible, and low-cost super-hydrophobic N-CNTs@SS cathodes for high-capacity, high-rate, and stable Li-air batteries. *Adv. Energy Mater.* 8, 1702242 (2018).
3. Shu, C. et al Highly reversible Li-O_2 battery induced by modulating local electronic structure via synergistic interfacial interaction between ruthenium nanoparticles and hierarchically porous carbon. *Nano Energy* 57, 166-175 (2019).
4. Guo, X. et al. 3D nanoporous nitrogen-doped graphene with encapsulated RuO_2 nanoparticles for Li-O_2 batteries. *Adv. Mater.* 27, 6137-6143 (2015).
5. Tu, F. et al. Au-decorated cracked carbon tube arrays as binder-free catalytic cathode enabling guided Li_2O_2 inner growth for high-performance Li-O_2 batteries. *Adv. Funct. Mater.* 26, 7725-7732 (2016).
6. Lin, X. et al. An open-structured matrix as oxygen cathode with high catalytic activity and large Li_2O_2 accommodations for lithium-oxygen batteries. *Adv. Energy Mater.* 8, 1800089 (2018).
7. Cai, S. et al. A synergistic catalytic mechanism for oxygen evolution reaction in aprotic Li-O_2 battery. *ACS Catal.* 8, 7983-7990 (2018).
8. Gong, Y. et al. Inverse spinel cobalt-iron oxide and N-doped graphene composite as an efficient and durable bifuctional catalyst for Li-O_2 batteries. *ACS Catal.* 8, 4082-4090 (2018).
9. Huang, Z. et al. A Li-O_2 battery cathode with vertical mass/charge transfer pathways. *J. Mater. Chem. A* 7, 3000-3005 (2019).
10. Cao, X. et al. Oxygen defect-ridden molybdenum oxide-coated carbon catalysts for Li-O_2 battery cathodes. *Appl. Catal., B* 253, 317-322 (2019).
11. Shen, J. et al. Biomass-derived hierarchically porous carbon skeletons with in situ decorated IrCo nanoparticles as high-performance cathode catalysts for Li-O_2 batteries. *J. Mater. Chem. A* 7, 10662-10671 (2019).
12. Jo, Y. K. et al. Multilayer hybrid nanosheet of mesoporous carbon-layered metal oxide as a highly efficient electrocatalyst for Li-O_2 batteries. *Appl. Catal., B* 254, 523-530 (2019).
13. Pham, H. T. T., Kim, Y., Kim, Y. J., Lee, J. W. & Park, M. S. Robust design of dual-phasic carbon cathode for lithium-oxygen batteries. *Adv. Funct. Mater.* 29, 1902915 (2019).
